# Antitumor Efficacy of Apatinib and Etoposide-Loaded D-α-Tocopheryl Polyethylene Glycol Succinate Mixed Micelles Against Multidrug-Resistant Ovarian Cancer Cells

**DOI:** 10.3390/pharmaceutics18091143

**Published:** 2026-09-10

**Authors:** Myeong Kyun Yoo, Su Jeong Kang, Min Jeong Jo, Jae Min Lee, Moon Sup Yoon, Seon Min Park, Sinem Yaprak Karavana, Dae Hwan Shin

**Affiliations:** 1 College of Pharmacy, Chungbuk National University, Cheongju 28160, Republic of Korea; 2 Department of Pharmaceutical Technology, Faculty of Pharmacy, Ege University, 35040 Bornova, Izmir, Turkey; 3 Korea Animal Replacement Experiment Co., Ltd. (KARE), Cheongju 28160, Republic of Korea; 4 Chungbuk National University Hospital, Chungbuk National University, Cheongju 28644, Republic of Korea

**Keywords:** ovarian cancer, multidrug resistance (MDR), Apatinib, etoposide, polymeric mixed micelles, D-α-tocopheryl polyethylene glycol succinate (TPGS), Soluplus^®^, P-glycoprotein-associated efflux, combination chemotherapy, synergistic therapy

## Abstract

**Background:** Ovarian cancer is frequently diagnosed at an advanced stage and remains one of the most lethal gynecologic malignancies. The development of multidrug resistance (MDR) during repeated chemotherapy is a major cause of treatment failure and is often associated with increased drug efflux mediated by transporters such as P-glycoprotein (P-gp). In this study, D-α-tocopheryl polyethylene glycol succinate (TPGS) and Soluplus^®^ (SOL) mixed micelles, abbreviated as TS, were developed to co-deliver apatinib (APA), a VEGFR-2 inhibitor, and etoposide (ETP), a topoisomerase II inhibitor, for MDR ovarian cancer models. The formulation was designed to improve the aqueous dispersion, cellular accumulation, and antitumor activity of APA and ETP. **Methods:** APA/ETP-loaded TS micelles (APA/ETP-mTS) were prepared and characterized in terms of particle size, polydispersity index (PDI), zeta potential, encapsulation efficiency (EE), storage stability, and in vitro drug release. Anticancer efficacy was assessed using MTT assays, cellular uptake assays, and 3D tumor spheroid studies employing HeyA8-MDR cells, followed by in vivo toxicity and antitumor efficacy studies. Micellar formulations were denoted using the cargo–carrier format, where APA/ETP indicates co-loaded APA and ETP, C6 indicates coumarin-6 (C6) used as a fluorescent probe, mTS indicates TPGS/SOL mixed micelles, and mSOL indicates SOL-only micelles. **Results:** The selected APA/ETP-mTS formulation showed a particle size of 20.0 ± 5.1 nm, a PDI of 0.16 ± 0.05, near-neutral zeta potential, and encapsulation efficiencies exceeding 60% for both drugs. The micelles maintained colloidal stability at 4 °C for 4 weeks, although partial decreases in encapsulation efficiency were observed. Compared with APA/ETP solution, APA/ETP-mTS delayed the release of both drugs. C6-mTS showed higher intracellular fluorescence intensity than C6-mSOL, suggesting enhanced cellular accumulation associated with TPGS incorporation. APA/ETP-mTS showed greater cytotoxicity than free drugs in HeyA8-MDR monolayer cells and produced the strongest spheroid growth inhibition among the tested micellar formulations. In the HeyA8-MDR xenograft model, APA/ETP-mTS suppressed tumor growth and resulted in the lowest final tumor weight without apparent overt toxicity based on body weight and survival observations. **Conclusions:** These results suggest that APA/ETP-mTS is a promising micellar co-delivery platform for hydrophobic anticancer drugs in MDR ovarian cancer.

## 1. Introduction

Ovarian cancer is often diagnosed at an advanced stage due to the absence of early symptoms and is characterized by high recurrence and mortality rates, making it one of the most challenging malignancies to treat [1,2,3]. Recent studies have further highlighted that ovarian cancer progression and therapeutic response are influenced by the complex tumor microenvironment and diverse molecular regulatory mechanisms associated with tumor growth and metastasis [4,5]. In particular, the development of multidrug resistance (MDR) during repeated chemotherapy cycles severely diminishes therapeutic efficacy and ultimately lowers patient survival [6]. MDR arises through multiple mechanisms, including the overexpression of drug efflux pumps such as P-glycoprotein (P-gp), alterations in drug targets, and the evasion of apoptosis, highlighting the urgent need for advanced drug delivery systems capable of addressing these resistance pathways [3,7,8,9].

Combination chemotherapy is widely used in cancer treatment to target multiple signaling pathways simultaneously, reduce the likelihood of drug resistance, and improve therapeutic efficacy compared with single-agent therapy. In particular, synergistic drug combinations can produce enhanced antitumor effects at lower individual drug doses, potentially reducing dose-related toxicity while improving tumor suppression. Therefore, identifying suitable drug combinations and developing delivery systems capable of co-delivering synergistic agents are important strategies for overcoming MDR-associated treatment failure.

Apatinib (APA) is a selective VEGFR-2 tyrosine kinase inhibitor (TKI) that suppresses tumor angiogenesis by blocking VEGF/VEGFR-2 signaling, thereby inhibiting tumor growth and metastatic progression [10,11]. As a TKI, APA also attenuates downstream survival pathways such as the PI3K/AKT/mTOR axis, contributing to enhanced antitumor activity [12]. Recent studies have suggested that APA may modulate P-gp–associated drug efflux in MDR cancer cells, indicating its potential utility as part of combination strategies aimed at improving intracellular drug retention [13,14,15]. However, the therapeutic impact of APA is limited by its poor aqueous solubility, low bioavailability, rapid clearance, and dose-dependent systemic toxicity, highlighting the need for an optimized drug delivery system to improve its pharmacokinetic profile and intracellular accumulation, particularly in MDR tumor environments [16,17].

Etoposide (ETP), a chemotherapeutic agent that inhibits DNA topoisomerase II, interferes with DNA replication and induces apoptosis [18,19]. Although widely used for various solid tumors, its efficacy is significantly reduced in MDR cells due to P-gp–mediated efflux [20]. Additionally, rapid systemic clearance and nonspecific toxicity limit its therapeutic application. These limitations can be addressed through nanoparticle-based co-delivery strategies [18,21,22].

Previous studies have reported that APA-based combination regimens, including combinations with ETP, can improve therapeutic responses in advanced solid tumors [23]. These findings support the rationale for evaluating APA and ETP as a combination pair in MDR cancer model. Mechanistically, APA has been reported to modulate P-gp-mediated drug efflux, which may enhance intracellular concentrations of chemotherapeutic agents and further support combination strategies with etoposide [24,25].

In this study, we developed a mixed micellar nanoformulation capable of co-loading APA and ETP using D-α-tocopheryl polyethylene glycol succinate (TPGS)–based micelles. TPGS has been reported to modulate P-gp-associated drug efflux, which may contribute to improved intracellular drug retention in MDR cancer cells; however, its relatively high critical micelle concentration (CMC) limits micellar stability under physiological dilution when used alone [26,27]. To address this limitation, Soluplus^®^ (SOL), an amphiphilic graft copolymer with an ultralow CMC, was incorporated into the formulation [28].

SOL possesses an extremely low CMC, enabling the micelles to remain intact even under substantial dilution in biological environments [29,30]. This characteristic significantly enhances the overall stability of the mixed micelles [31]. In addition, SOL improves the solubility of poorly water-soluble drugs and exhibits favorable interfacial properties, thereby increasing drug loading capacity and EE [28,32].

The combined TPGS/SOL mixed micelles (TS) were designed to leverage the ultralow-CMC stability of SOL together with the reported P-gp-modulatory properties of TPGS, enabling co-loading of APA and ETP, improved physicochemical stability, and potentially enhanced intracellular drug accumulation in MDR cancer cells [32,33].

**Figure 1:**
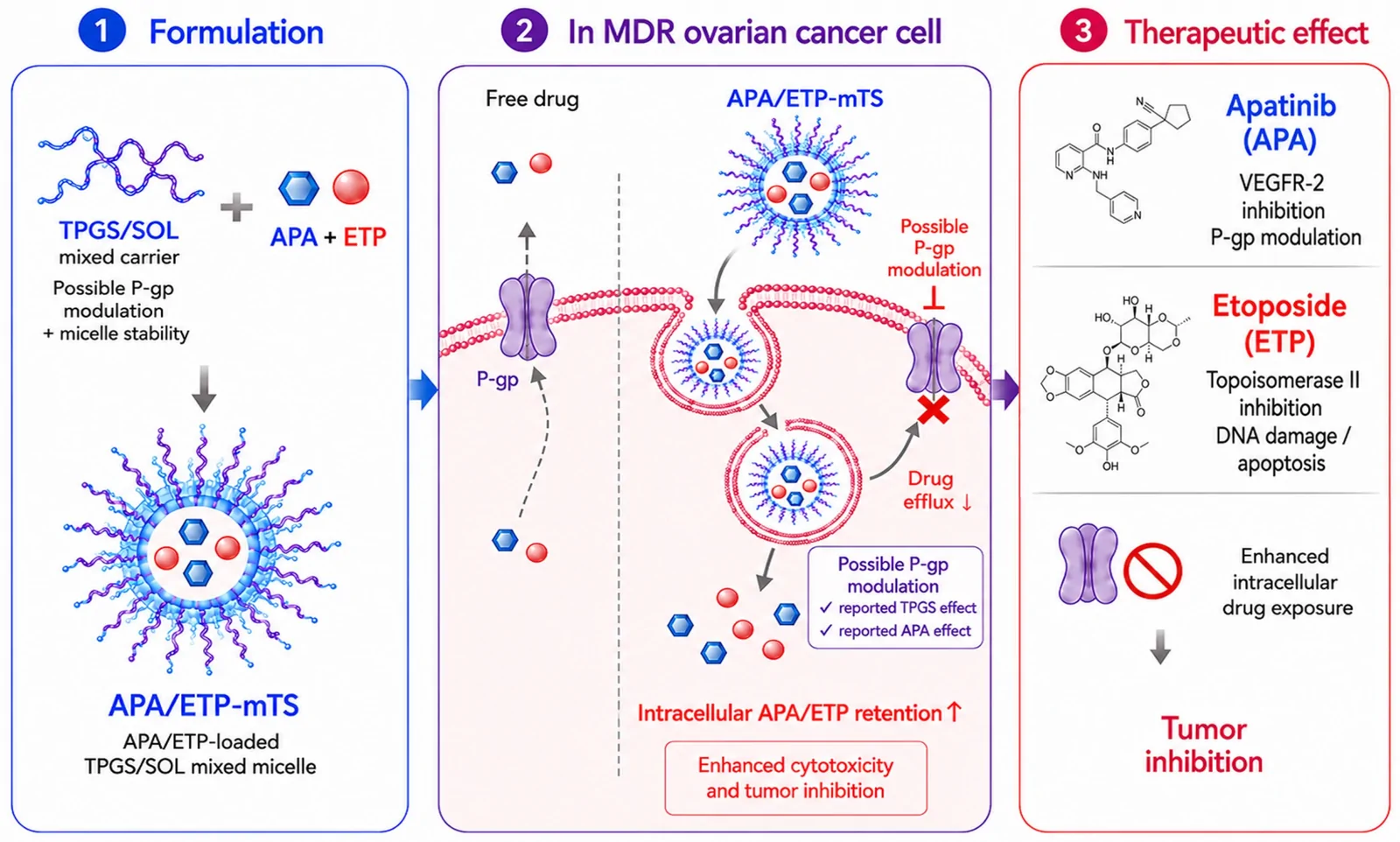
Schematic illustration of TPGS and Soluplus^®^ mixed micelles encapsulating APA and ETP, and their proposed mechanism in MDR ovarian cancer cells.

In this work, we prepared APA/ETP-mTS and evaluated their potential as a co-delivery system for MDR ovarian cancer treatment (Figure 1). The formulation was assessed in terms of physicochemical properties, storage stability, in vitro drug release, cytotoxicity in MDR ovarian cancer cells, cellular uptake, 3D tumor spheroid growth inhibition, systemic tolerability, and antitumor efficacy in a subcutaneous xenograft model.

## 2. Materials and Methods

### 2.1. Materials

Apatinib was purchased from Selleckchem (Houston, TX, USA), and etoposide was obtained from Tokyo Chemical Industry, TCI (Tokyo, Japan). TPGS, coumarin-6 (C6), thiazolyl blue tetrazolium bromide (MTT), tert-butanol, Triton X-100, sodium hydroxide (NaOH) solution, Cremophor EL^®^, and dimethyl sulfoxide (DMSO) were acquired from Sigma-Aldrich (Merck, St. Louis, MO, USA). SOL was kindly provided by BASF (Ludwigshafen, Germany). Methanol (MeOH) was purchased from Honeywell Burdick & Jackson (Muskegon, MI, USA), and acetonitrile was obtained from Thermo Fisher Scientific (Waltham, MA, USA). High-purity distilled water (DW) was supplied by Tedia (Fairfield, OH, USA). All other chemicals and reagents used in this study were of analytical grade.

### 2.2. Methods

#### 2.2.1. Cell Lines and Culture Conditions

Human ovarian cancer HeyA8 cells were kindly provided by Dr. Anil K. Sood (Department of Cancer Biology, The University of Texas MD Anderson Cancer Center). The paclitaxel-resistant human ovarian cancer cell line HeyA8-MDR was provided by Dr. Jeong-Won Lee (Samsung Medical Center, Seoul, Republic of Korea). In a previous report from Dr. Lee’s group, HeyA8 and HeyA8-MDR cells were described as human epithelial ovarian cancer cell lines originally obtained from Dr. Anil K. Sood at the Department of Cancer Biology, The University of Texas MD Anderson Cancer Center, Houston, TX, USA, supporting the documented common provenance of these related cell lines [34]. Both cell lines were cultured in RPMI-1640 medium (Roswell Park Memorial Institute formulation) and maintained in an incubator at 37 °C with 5% CO_2_. The culture medium was supplemented with 1% (*v*/*v*) penicillin–streptomycin and 10% (*v*/*v*) fetal bovine serum. To preserve the MDR phenotype, HeyA8-MDR cells were exposed to 300 ng/mL paclitaxel [35,36,37]. To minimize potential interference from paclitaxel, HeyA8-MDR cells were cultured in paclitaxel-free medium for one passage before each experiment. All subsequent drug-response assays, including MTT, cellular uptake, and spheroid assays, were performed in the absence of paclitaxel. All culture-related reagents and consumables were obtained from Corning, Inc. (Corning, NY, USA).

#### 2.2.2. High-Performance Liquid Chromatography Analysis

The concentrations of APA and ETP were analyzed using a Waters HPLC system (Milford, MA, USA) equipped with a 2996 photodiode array detector and a 2695 separation module. A Fortis C18 chromatographic column (5 µm, 4.6 × 250 mm; Fortis Technologies Ltd., Cheshire, UK) was used, and the column temperature was maintained at 30 °C. The injection volume for both APA and ETP was 10 µL. The mobile phase consisted of ACN/DW (65:35, *v*/*v*) with 0.1% (*v*/*v*) triethylamine (TEA) and was eluted isocratically at a flow rate of 1.0 mL/min. APA and ETP were detected at wavelengths of 260 nm and 284 nm, respectively [38,39].

#### 2.2.3. Evaluating the Synergistic Effect of APA and ETP in HeyA8-MDR

To evaluate the interaction between APA and ETP, HeyA8-MDR cells were treated with APA and ETP either individually or in combination at predefined molar ratios. Cell viability was determined using the MTT assay after 48 h of treatment, and IC_50_ values were calculated from dose–response curves. Drug–drug interactions were evaluated using the Combination Index (CI) method developed by Chou (2010) [40]. The CI value was calculated according to the following equation:

$$Combination\;Index\left(CI\right)=\frac{{\left(D\right)}_{1}}{{\left(D_{x}\right)}_{1}}+\frac{{\left(D\right)}_{2}}{{{(D}_{x})}_{2}}$$

where $(D_{x}{)}_{1}$ and $(D_{x}{)}_{2}$ represent the individual IC_50_ values of the first and second drugs, respectively, and $(D{)}_{1}$ and $(D{)}_{2}$ represent the corresponding IC_50_ values when the two drugs were administered in combination. A CI value greater than 1 indicates antagonism, a value equal to 1 indicates additivity, and a value less than 1 indicates synergism. In this study, the interaction between APA and ETP was assessed using this same CI analysis approach.

#### 2.2.4. Formulation and Physicochemical Characterization of TPGS Mixed Micelles

TS loaded with APA and ETP were prepared using thin-film hydration method [41].

For the thin-film hydration method, accurately weighed amounts of APA, ETP, TPGS, and SOL were dissolved in 1 mL of methanol and transferred into a round-bottom flask. The solvent was evaporated under vacuum using a rotary evaporator (EYELA^®^, Bohemia, NY, USA) in a 60 °C water bath for 10 min. After a uniform thin film was formed, 1.2 mL of PBS (pH 7.4) was added, followed by hydration for 30 min to obtain the TS micellar solution.

Prepared micelle samples were centrifuged at 13,000 rpm for 5 min at 4 °C (Hanil Sciencific Inc., Gimpo, Republic of Korea) to collect the supernatant and subsequently filtered through a sterile 0.2 μm membrane filter (Sartorius, Göttingen, Germany). The physicochemical properties, including particle size, polydispersity index (PDI), and zeta potential, were measured by dynamic light scattering (DLS) using an instrument (Litesizer 500, Anton Paar, Graz, Austria) at 25 °C under automatic scattering angle conditions. For accurate measurements, samples were diluted 10-fold with DW before measurement.

Encapsulation efficiency (EE, %) was calculated as follows:

EE (%) = (amount of drug encapsulated in micelles/initial amount of drug added) × 100.

Drug loading capacity (DL, %) was calculated as follows:

DL (%) = (amount of drug encapsulated in micelles/total amount of drug-loaded micelles) × 100.

Drug-loaded micelles were denoted using the drug–micelle format. TPGS/SOL mixed micelles were abbreviated as mTS, whereas SOL-only micelles were abbreviated as mSOL. Thus, APA/ETP-mTS and APA/ETP-mSOL refer to APA/ETP-loaded TPGS/SOL mixed micelles and SOL-only micelles, respectively. C6-loaded micelles used for cellular uptake studies were denoted as C6-mTS and C6-mSOL.

To visualize the morphology of APA/ETP-mTS, transmission electron microscopy (TEM) (JEM-2100, JEOL Ltd., Tokyo, Japan) was employed. Sample preparation for TEM involved depositing a diluted micelle solution onto the center of a copper grid coated with a 200-mesh formvar carbon film. The grid was then air-dried and left to stabilize at 25 °C for 2 days. Subsequently, the treated samples were observed using TEM at an acceleration voltage of 200 kV.

#### 2.2.5. In Vitro Storage Stability Test

The stability of APA/ETP-mTS was evaluated at 4 °C (refrigeration) and 37 °C (water bath). Samples were collected on days 0, 1, 2, 4, 7, 14, and 28. Each sample was diluted 1:10 with distilled water, after which particle size, PDI, and zeta potential were measured using a DLS instrument, and encapsulation efficiency (EE, %) was determined using HPLC. All experiments were performed in triplicate.

#### 2.2.6. In Vitro Drug Release Assay

The release profiles of APA and ETP from APA/ETP solution and micellar formulations were evaluated using the dialysis method [42]. Samples of APA/ETP solution, APA-mTS, ETP-mTS, or APA/ETP-mTS were loaded into dialysis bags with a molecular weight cutoff of 20 kDa and immersed in 2.0 L of phosphate-buffered saline (PBS, pH 7.4). The APA/ETP solution was prepared using a mixture composed of 30% Cremophor EL^®^, 20% ethanol, and 50% distilled water. The experiment was conducted at 37 °C with continuous stirring at 200 rpm using a magnetic stirrer. Samples were collected at predetermined time points (0, 2, 4, 6, 8, 24, 48, 72, 168, 240, and 336 h). Each collected aliquot was diluted tenfold with acetonitrile and analyzed by HPLC. To minimize drug saturation in the release medium, the PBS release medium was replaced at 8, 24, 72, 168, and 240 h. All experiments were performed in triplicate, and the drug release profiles were analyzed using Korsmeyer–Peppas kinetic model fitting with SigmaPlot (Systat Software, Inc., San Jose, CA, USA). Korsmeyer–Peppas release exponent (*n*) was used to support interpretation of the release mechanism. The Korsmeyer–Peppas model was expressed as *F = k·t^n^*, where F is the cumulative release percentage at time *t, k* is the release rate constant, and *n* is the release exponent. An *n* value below 0.43 was interpreted as diffusion-controlled release.

#### 2.2.7. In Vitro Cytotoxicity Assay

Cytotoxicity was evaluated using the MTT assay [43,44]. HeyA8 cells and HeyA8-MDR cells were seeded into 96-well plates at a density of 4000 cells per well (n = 6 wells) and incubated for 24 h at 37 °C in a humidified atmosphere containing 5% CO_2_. After incubation, cells were treated with various molar ratios of APA and ETP solutions, as well as APA-mTS, ETP-mTS, or APA/ETP-mTS formulations. Untreated cells were used as the control group. Following 48 h of treatment, the culture medium was carefully removed, and 100 µL of MTT solution (0.5 mg/mL) was added to each well. After a 4 h incubation period, the MTT solution was discarded, and 100 µL of DMSO was added to dissolve the formazan crystals. The plates were gently shaken for 10 min at 200 rpm using an orbital shaker (NB-101S; N-BIOTEK, Bucheon, Republic of Korea) to ensure complete dissolution. The optical density of each well was measured at 540 nm using a microplate reader (SpectraMax ID3; Molecular Devices, San Jose, CA, USA). All data analysis was performed using GraphPad Prism v8.4.2 (GraphPad Software, La Jolla, CA, USA).

#### 2.2.8. In Vitro Cellular Uptake Assay

C6, a hydrophobic fluorescent probe, was used as a model cargo to evaluate cellular uptake of the micellar formulations. For quantitative fluorescence analysis, HeyA8-MDR cells were seeded into black 96-well plates at a density of 2 × 10^5^ cells per well. After the cells reached confluence, 100 µL of either C6-mSOL, or C6-mTS—each containing the same concentration of C6—was added to the wells. For comparison, C6-mSOL was used as a TPGS-free micellar control to evaluate the contribution of TPGS incorporation. The incubation times were set to 1, 2, 4, 6, and 8 h. At each time point, the culture medium was carefully removed, and the wells were washed three times with 50 µL of PBS. Subsequently, 50 µL of 0.5% Triton X-100 prepared in 0.2 N NaOH was added to lyse the cells. The fluorescence intensity in each well was measured using a microplate reader, with excitation and emission wavelengths set to 460 nm and 505 nm, respectively [42,45].

#### 2.2.9. In Vitro Cytotoxicity Assay Using HeyA8-MDR Tumor Spheroids

Cancer cells are known to exhibit high proliferative capacity and self-renewal characteristics. Based on previously reported methods, HeyA8-MDR tumor spheroids were generated for this study [46,47]. Briefly, 7.5 µL of Matrigel (Corning, NY, USA) was mixed with 7.5 µL of a cell suspension (1.33 × 10^4^ cells/µL) at a 1:1 ratio and dispensed onto an ultra-low attachment (ULA) 100 mm culture dish. After adding RPMI-1640 medium, the cells were incubated at 37 °C for 24 h to initiate spheroid formation. The culture dish was then transferred to an NB-101SRC orbital shaker (N-BIOTEK, Bucheon, Republic of Korea) and maintained for approximately two weeks until well-formed tumor spheroids were obtained. The morphological characteristics of the spheroids were examined using an optical microscope.

To selectively stain live HeyA8-MDR cells, a Live/Dead Cell Imaging Kit (Thermo Fisher Scientific, Waltham, MA, USA) was employed. Fluorescence signals corresponding to live cells within the tumor spheroids were measured at days 0, 2, 4, 7, and 10 using an in vivo imaging system (IVIS; VISQUE InVivo Smart-LF, Anyang, Republic of Korea). Viable cells within the tumor spheroids were quantified using CleVue™ software (Vieworks, Anyang, Republic of Korea). For cytotoxicity evaluation, HeyA8-MDR spheroids were treated with APA-mTS, ETP-mTS, or APA/ETP-mTS at equivalent drug concentrations every two days. For the control group, only fresh culture medium was added at the same time points without drug administration.

#### 2.2.10. In Vivo Toxicity Test

All animal experiments and procedures were approved by the Institutional Animal Care and Use Committee (IACUC) of Chungbuk National University (Approval No.: CBNUA-25-0103-01, approved on 16 September 2025), and all studies were conducted in accordance with the guidelines of the Institute of Laboratory Animal Resources (ILAR).

In this study, six-week-old female ICR mice were purchased from OrientBio Inc. (Seongnam, Republic of Korea). The mice were randomly assigned to eight groups (n = 6 per group). During the experimental period, animals were provided with free access to food and water and were allowed a one-week acclimation period prior to treatment. Intravenous administration was performed every other day for two weeks.

The treatment groups were organized as follows: control, blank mTS (drug-free mTS), APA-mTS (5 and 10 mg/kg), ETP-mTS (5 and 10 mg/kg), and APA/ETP-mTS (5/5 and 10/10 mg/kg).

Body weight was measured every two days, with the initial body weight considered as 100%. Toxicity attributable to the formulations was defined as a body weight loss exceeding 20%, abnormal behavior, or mortality [48,49].

#### 2.2.11. In Vivo Antitumor Efficacy

To establish the xenograft model, five-week-old female BALB/c nude mice were purchased from RaonBio (Yongin-si, Republic of Korea).

For subcutaneous implantation, HeyA8-MDR cells were suspended in DPBS and mixed with Matrigel at a 1:1 ratio (*v*/*v*). A total volume of 200 µL containing 2.0 × 10^6^ cells was injected subcutaneously [50]. Treatment was initiated 7 days after tumor inoculation, and this time point was defined as treatment day 0. Mice were randomly assigned into four groups to evaluate antitumor efficacy: control, APA-mTS, ETP-mTS, and APA/ETP-mTS. Drug treated groups received APA (10 mg/kg) and ETP (10 mg/kg), administered via tail-vein injection every other day, while the control group received no treatment. Tumor volume (V) was measured using calipers and calculated according to standard formulas [51].

$$V=\frac{\left(Length\right)\times {\left(Width\right)}^{2}}{2}$$

Mice were monitored every two days, and all animals were sacrificed three weeks after tumor inoculation. On the day of sacrifice, body weight and tumor weight were recorded, and tumors were photographed for documentation.

#### 2.2.12. Tumor H&E Staining

Tumor slides were prepared for H&E staining according to previously reported procedures [52,53]. Slide images were acquired using a Pannoramic SCAN II slide scanner (3DHISTECH, Budapest, Hungary) at 20× magnification. The images were subsequently processed and analyzed using CaseViewer software version 2.7 (3DHISTECH).

#### 2.2.13. Statistical Analysis

Experiments were performed at least in triplicate, and data are presented as mean ± standard deviation. Statistical analysis was performed using unpaired *t*-tests with GraphPad Prism v8.4.2 software. For comparisons involving more than two groups, one-way or two-way analysis of variance (ANOVA) was performed as appropriate, followed by post hoc multiple-comparison tests. For longitudinal or repeated time-course datasets, two-way ANOVA was used to evaluate the effects of treatment, time, and their interaction, followed by multiple-comparison correction. Statistical significance was defined as follows: * *p* < 0.05, ** *p* < 0.01, *** *p* < 0.001, and **** *p* < 0.0001

## 3. Results

### 3.1. Comparative Cytotoxicity of HeyA8 and HeyA8-MDR Cells

To assess the drug-resistant characteristics of HeyA8-MDR cells, cytotoxicity assays were performed in both parental HeyA8 and HeyA8-MDR cells using free APA and ETP. HeyA8-MDR cells showed lower sensitivity to both drugs than parental HeyA8 cells (Figure S1). The IC_50_ value of APA in HeyA8-MDR cells was approximately 4.93-fold higher than that in HeyA8 cells (*** *p* < 0.001), while the IC_50_ value of ETP was approximately 6.40-fold higher (*** *p* < 0.001). These quantitative differences indicate that HeyA8-MDR cells retained a resistant phenotype under the present culture conditions [54].

### 3.2. Evaluating the Synergistic Effect of APA and ETP in HeyA8-MDR Cells

**Figure 2:**
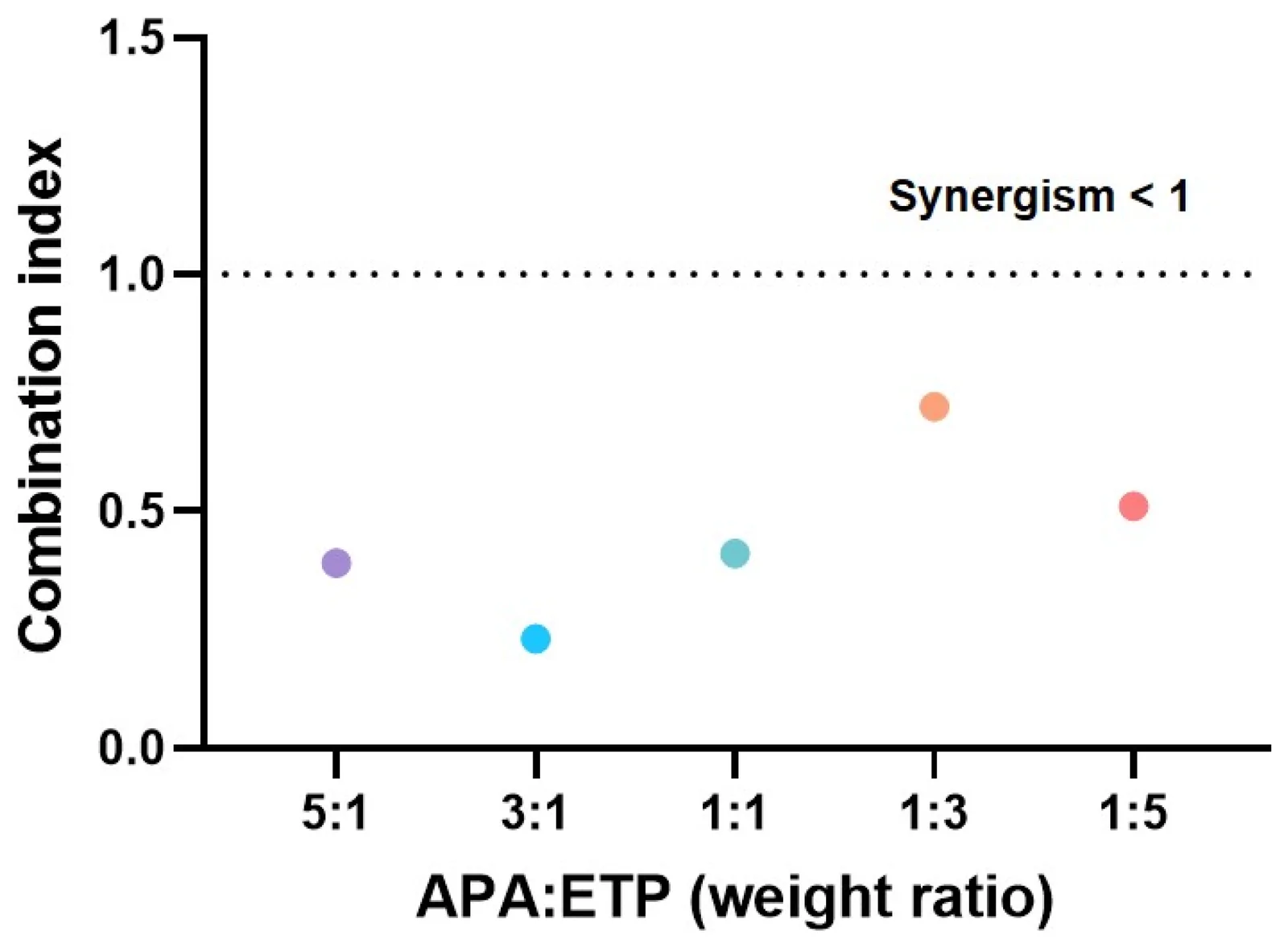
Combination index (CI) values of APA and ETP combinations in HeyA8-MDR cells at the IC_50_ level. APA and ETP were tested at predefined weight ratios of 5:1, 3:1, 1:1, 1:3, and 1:5, corresponding to APA:ETP molar ratios of 7.39:1, 4.43:1, 1.48:1, 1:2.03, and 1:3.38, respectively. CI < 1 indicates synergism. Data are presented as calculated CI values at the IC_50_ level.

To evaluate the interaction between APA and ETP in HeyA8-MDR cells, the two drugs were tested at predefined weight ratios of 5:1, 3:1, 1:1, 1:3, and 1:5, corresponding to APA:ETP molar ratios of 7.39:1, 4.43:1, 1.48:1, 1:2.03, and 1:3.38, respectively. IC_50_ and CI values were then calculated based on the corresponding molar ratios. As shown in Figure 2, all tested combinations showed CI values below 1 at the IC_50_ level, suggesting synergistic interactions between APA and ETP under the tested conditions [40]. Although the 4.43:1 molar ratio, corresponding to a 3:1 weight ratio, exhibited the lowest CI value, the 1.48:1 molar ratio, corresponding to a 1:1 weight ratio, also showed clear synergism and was selected for micelle preparation based on its compatibility with the intended co-loading composition and subsequent in vivo dosing regimen. This ratio was therefore used for the preparation of APA/ETP-mTS in the following experiments.

### 3.3. Physicochemical Characterization of TPGS/SOL Mixed Micelles

APA/ETP-mTS were prepared using thin-film hydration with a rotary evaporator method, and their physicochemical properties were compared at the selected APA:ETP molar ratio. As summarized in Table 1, the particle size, PDI, zeta potential, and EE values varied depending on the mTS polymer composition.

Among the formulations prepared by thin-film hydration, the formulation containing 100 mg of total polymer at a TPGS:SOL weight ratio of 3:1 showed a nanoscale particle size of 20.0 ± 5.1 nm, a narrow size distribution with a PDI of 0.16 ± 0.05, and a near-neutral zeta potential of −0.93 ± 0.97 mV. The relatively small size of APA/ETP-mTS may be attributed to the formation of compact mixed micelles through the combined TPGS/SOL composition, the high polymer-to-drug ratio, and the thin-film hydration process. Although APA/ETP-mTS was smaller than many conventional polymeric micelles, the low PDI values indicated a narrow size distribution. Representative TEM imaging provided qualitative morphological support for the formation of nanosized micellar structures, consistent with the DLS-based size analysis (Figure S2). The EE values of APA and ETP were 63.5 ± 0.6% and 65.2 ± 1.2%, respectively, indicating successful co-encapsulation of both drugs.

The thin-film hydration formulation with a TPGS:SOL weight ratio of 3:1 provided a balanced physicochemical profile, including small particle size, low PDI, near-neutral zeta potential, and acceptable encapsulation efficiencies for both APA and ETP. Therefore, this formulation was selected for subsequent characterization and biological evaluation.

### 3.4. In Vitro Storage Stability Test

**Figure 3:**
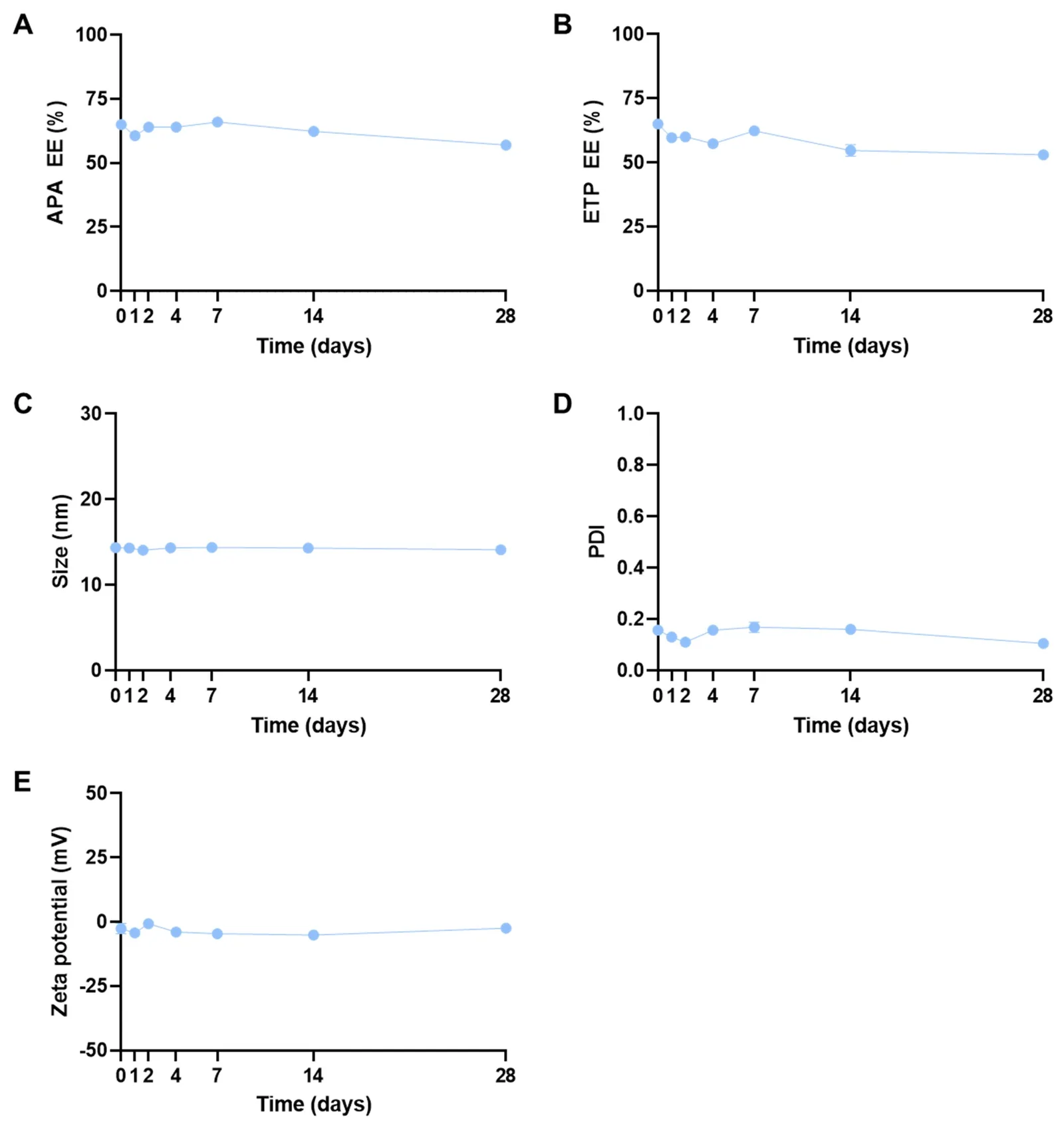
Stability evaluation of APA/ETP-mTS at 4 °C: (**A**) APA EE(%), (**B**) ETP EE(%), (**C**) particle size, (**D**) PDI, and (**E**) zeta potential (n = 3; Mean ± SD).

The storage stability of APA/ETP-mTS was evaluated for 4 weeks at 4 °C by monitoring EE, particle size, PDI, and zeta potential (Figure 3). The day 0 particle size was measured using an independently prepared batch of the selected APA/ETP-mTS formulation. The EE values of APA and ETP were 64.1 ± 1.24% and 65.4 ± 0.13% on day 0, respectively, and decreased to 57.0 ± 0.01% and 53.0 ± 0.01% by day 28. In contrast, the particle size remained essentially unchanged from 14.4 ± 0.06 nm on day 0 to 14.1 ± 0.09 nm on day 28, and the PDI showed only minor variation from 0.16 ± 0.01 to 0.11 ± 0.01. The zeta potential was changed from −2.63 ± 2.04 mV on day 0 to −2.43 ± 1.90 mV on day 28. These results indicate that APA/ETP-mTS micelles maintained good colloidal stability at 4 °C, with no substantial change in surface charge, although partial loss of drug encapsulation occurred during prolonged storage [55,56] (** *p* < 0.01, *** *p* < 0.001).

In an additional elevated-temperature stability assessment at 37 °C, APA/ETP-mTS showed particle size enlargement, increased PDI, and changes in EE values within the early storage period (Figure S3). These results suggest reduced formulation stability at 37 °C and further support refrigerated storage as the preferred condition for APA/ETP-mTS.

### 3.5. In Vitro Drug Release Assay

**Figure 4:**
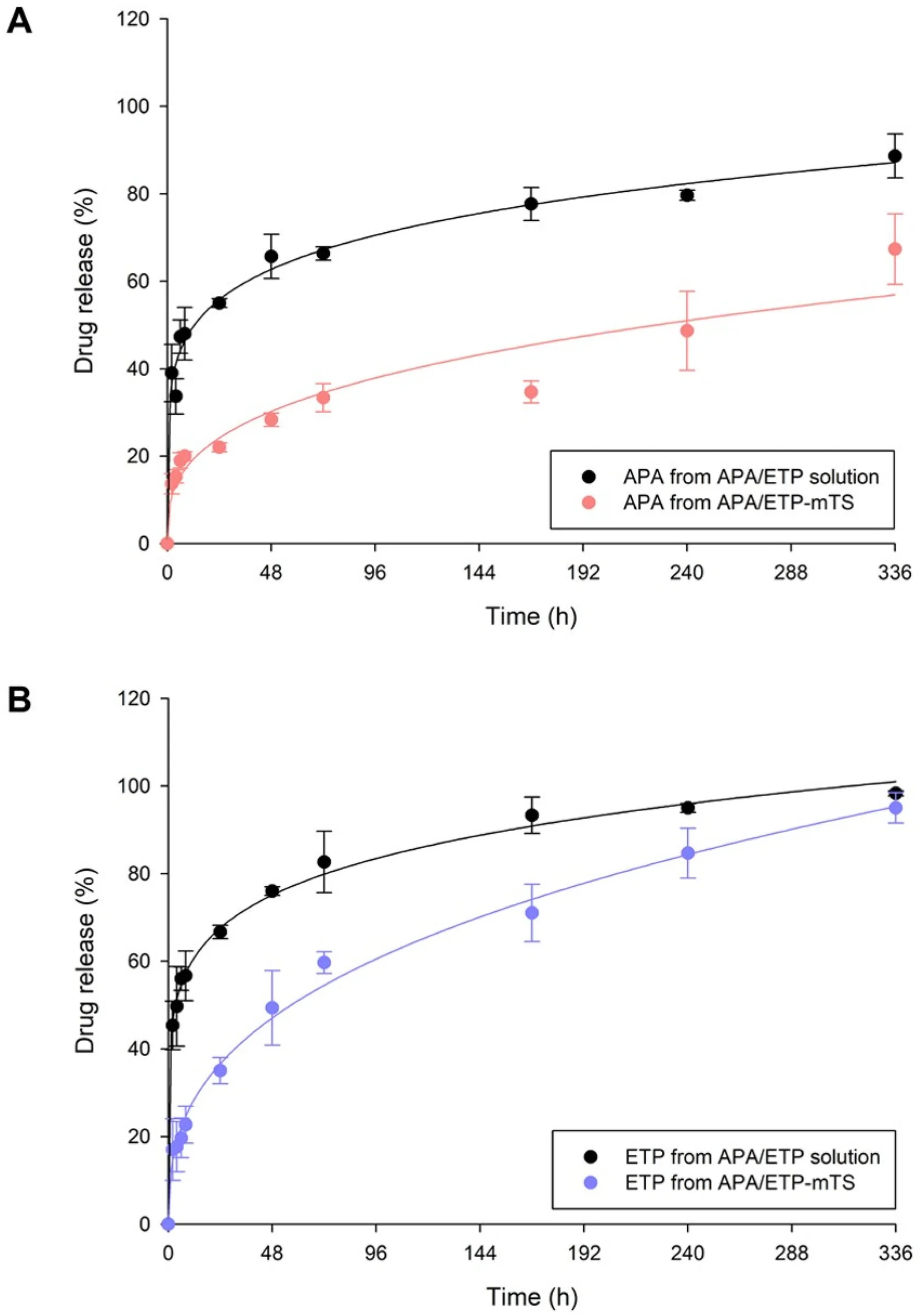
In vitro drug release profiles of APA and ETP from APA/ETP solution and TS micellar formulations. (**A**) Cumulative APA release from APA/ETP solution, and APA/ETP-mTS. (**B**) Cumulative ETP release from APA/ETP solution, and APA/ETP-mTS. (n = 3; Mean ± SD).

The in vitro release profiles of APA and ETP were evaluated using APA/ETP solution and APA/ETP-mTS (Figure 4). Both drugs showed formulation-dependent release behavior.

For APA, the APA/ETP solution showed relatively rapid release, reaching 47.3 ± 3.79% at 6 h, 66.3 ± 1.53% at 72 h, 77.7 ± 3.79% at 168 h, and 88.7 ± 5.03% at 336 h. In contrast, APA release from APA/ETP-mTS was delayed, with cumulative release values of 19.0 ± 1.73%, 33.3 ± 3.21%, 34.7 ± 2.52%, and 67.3 ± 8.08% at 6, 72, 168, and 336 h, respectively.

For ETP, the APA/ETP solution also showed faster release than APA/ETP-mTS, with cumulative release values of 56.0 ± 2.65% at 6 h, 82.7 ± 7.02% at 72 h, 93.3 ± 4.16% at 168 h, and 98.3 ± 0.58% at 336 h. In APA/ETP-mTS, ETP release was 19.7 ± 4.51%, 59.7 ± 2.52%, 71.0 ± 6.56%, and 95.0 ± 3.46% at 6, 72, 168, and 336 h, respectively.

Overall, APA release from the APA/ETP solution exceeded 60% by 72 h, whereas APA release from APA/ETP-mTS reached 60% only by 336 h. For ETP, both formulations reached approximately 60% release by 72 h, although APA/ETP-mTS showed lower release during the early phase. These results indicate delayed drug release from APA/ETP-mTS, particularly for APA.

To further evaluate the release mechanism, the release profiles of APA and ETP were fitted using the Korsmeyer–Peppas model (Table 2). All fitted release exponent (n) values were below 0.43, suggesting diffusion-controlled release behavior. Compared with APA/ETP solution, APA/ETP-mTS showed lower k values for both APA and ETP, decreasing from 32.6 to 8.61 for APA and from 41.6 to 11.5 for ETP. These kinetic results further support delayed drug release from APA/ETP-mTS while indicating that the release behavior remained primarily diffusion-controlled.

### 3.6. In Vitro Cytotoxicity Assay

**Figure 5:**
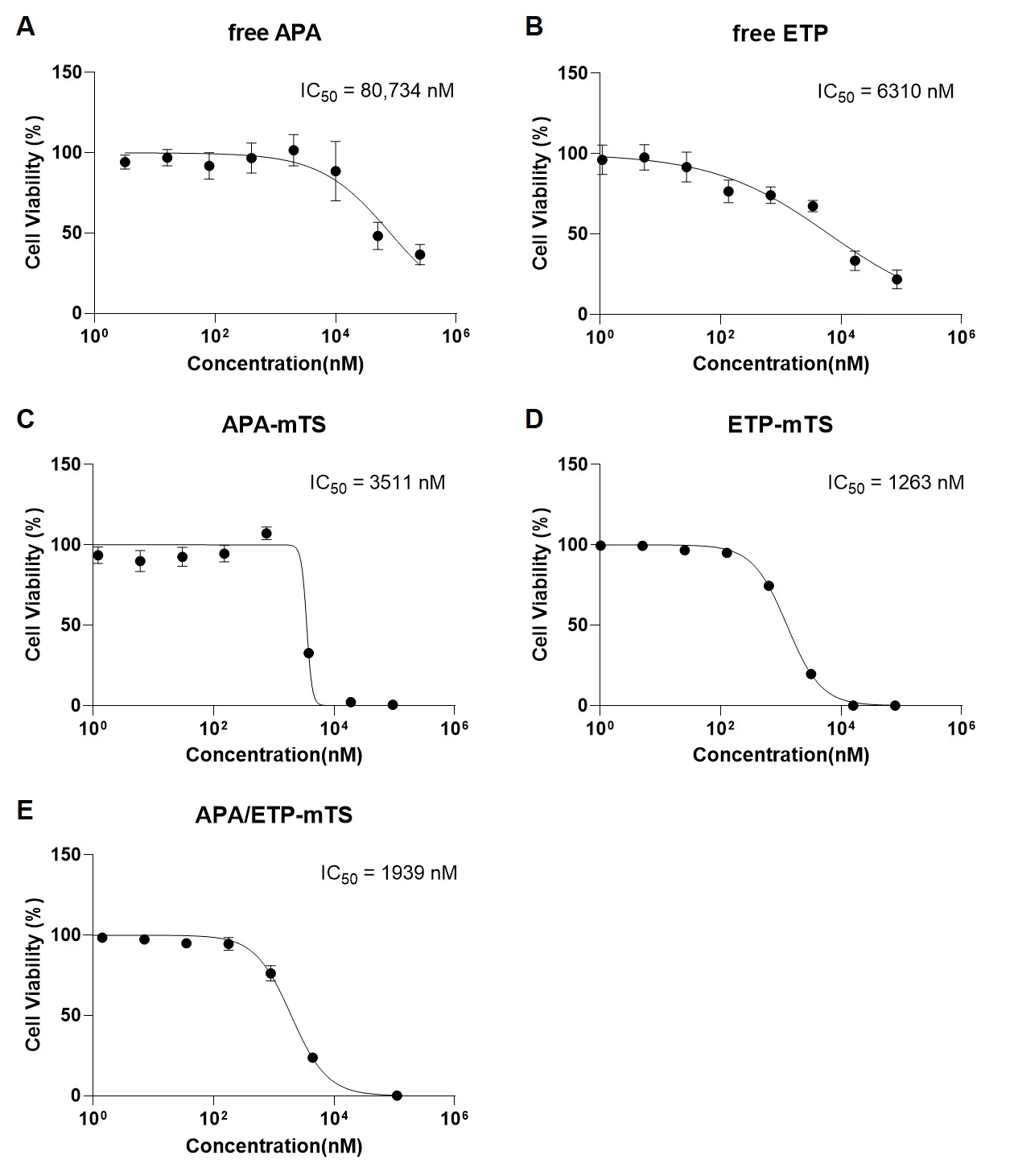
In vitro cytotoxicity of free drugs and micellar formulations in HeyA8-MDR cells. Dose–response curves of (**A**) free APA, (**B**) free ETP, (**C**) APA-mTS, (**D**) ETP-mTS, and (**E**) APA/ETP-mTS. (n = 6; Mean ± SD).

The cytotoxicity of free drugs and micellar formulations was evaluated in HeyA8-MDR cells using the MTT assay (Figure 5). The IC_50_ values of free APA and free ETP were 80,734 nM and 6310 nM, respectively, indicating that HeyA8-MDR cells were less sensitive to APA than to ETP under the tested conditions. In comparison, the IC_50_ values of APA-mTS, ETP-mTS, and APA/ETP-mTS were 3511 nM, 1263 nM, and 1939 nM, respectively.

Among the tested formulations, APA/ETP-mTS exhibited substantially greater cytotoxicity than free APA, and free ETP. These findings suggest that incorporation of TPGS into the mixed micelle formulation contributed to enhanced cytotoxicity in HeyA8-MDR cells, potentially by improving intracellular drug availability and modulating P-gp-associated drug efflux [57].

### 3.7. In Vitro Cellular Uptake Assay

**Figure 6:**
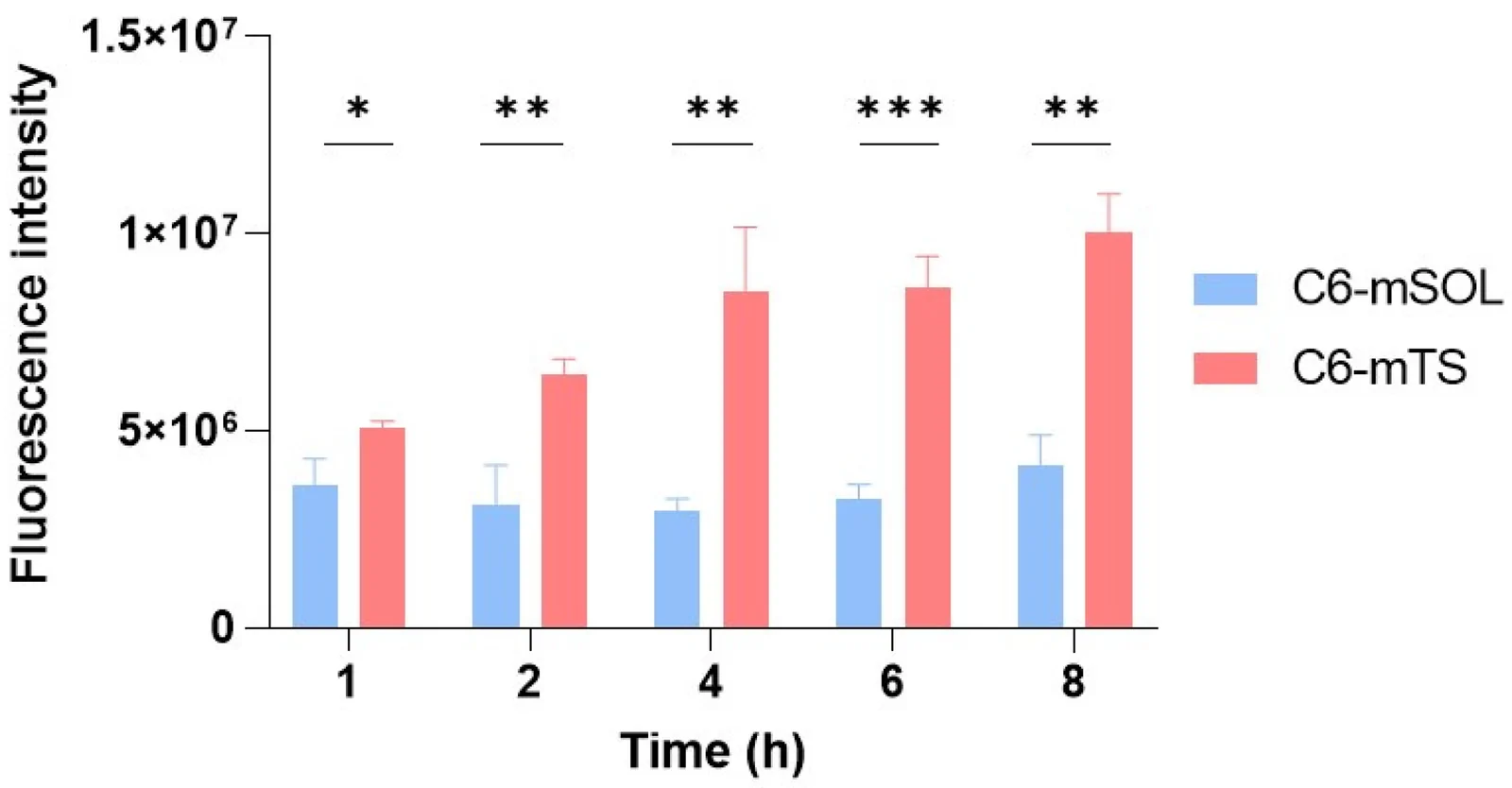
Cellular uptake of C6 in HeyA8-MDR cells after incubation with C6-mSOL and C6-mTS at equivalent concentrations. (n = 3; Mean ± SD).

Cellular accumulation of C6-loaded micelles in HeyA8-MDR cells was evaluated by measuring fluorescence intensity over time (Figure 6). C6-mTS showed higher fluorescence intensity than C6-mSOL at all measured time points. After 1 h of incubation, the fluorescence intensities were 3.61 × 10^6^ ± 5.27 × 10^5^ for C6-mSOL and 5.09 × 10^6^ ± 1.03 × 10^5^ for C6-mTS. After 8 h, the fluorescence intensity increased to 4.15 × 10^6^ ± 5.66 × 10^5^ for C6-mSOL and 1.00 × 10^7^ ± 7.51 × 10^5^ for C6-mTS. The significantly higher fluorescence intensity observed in the C6-mTS group suggests that TPGS incorporation was associated with enhanced cellular accumulation of C6 in HeyA8-MDR cells [58] (* *p* < 0.05, ** *p* < 0.01, *** *p* < 0.001).

### 3.8. In Vitro Cytotoxicity Assay Using HeyA8-MDR Tumor Spheroids

**Figure 7:**
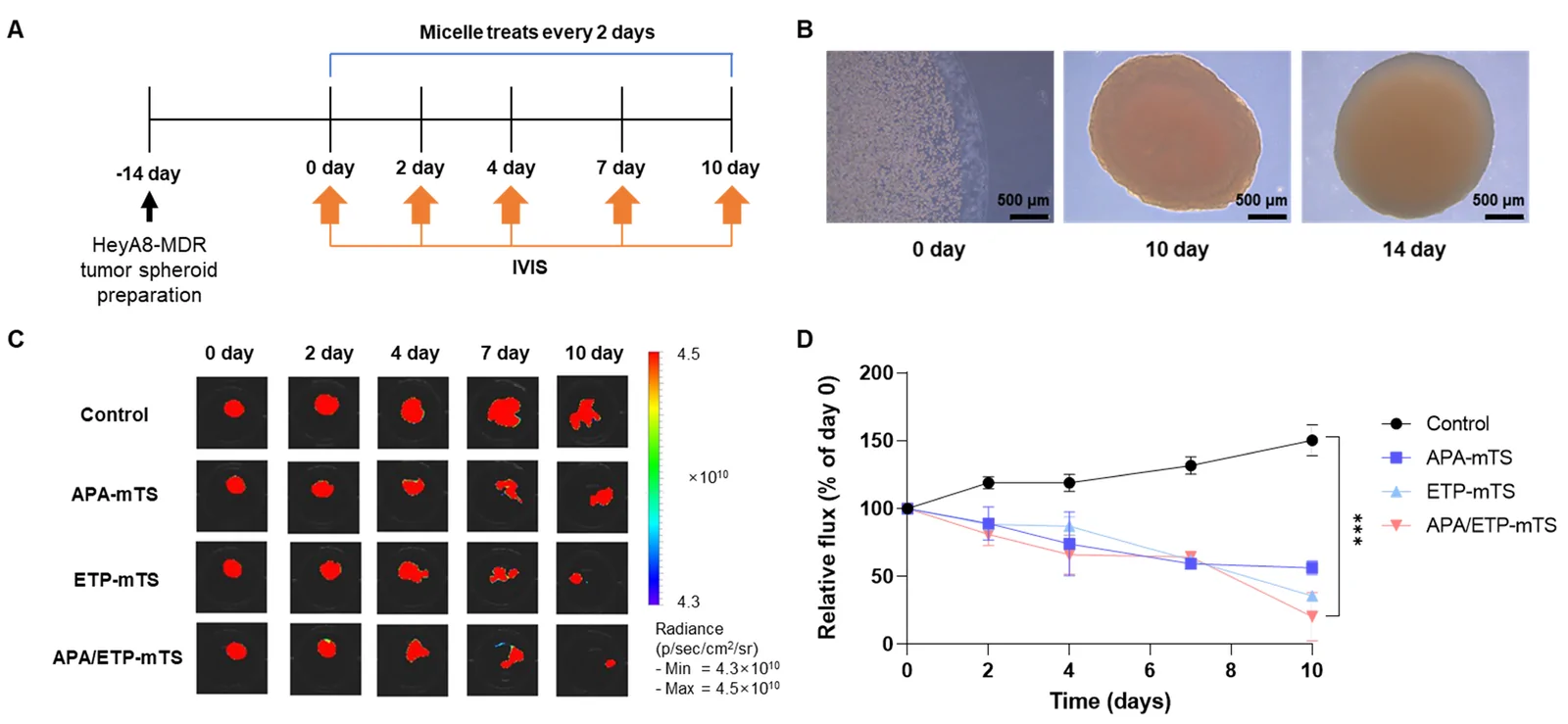
In vitro cytotoxicity assay using HeyA8-MDR tumor spheroids. (**A**) Experimental timeline for cytotoxicity evaluation using HeyA8-MDR tumor spheroids. (**B**) Representative microscopy images of spheroids during formation on culture days 0, 10, and 14. (**C**) Representative IVIS images of spheroids treated with micellar formulations. (**D**) Quantitative analysis of total flux values (*p*/sec/cm^2^/sr) on each day compared with day 0. (*** *p* < 0.001), (n = 3; Mean ± SD).

HeyA8-MDR tumor spheroids were generated and cultured for 14 days to obtain well-formed spheroids before drug treatment. During spheroid formation, morphological changes were monitored by microscopy on culture days 0, 10, and 14. After spheroid formation, the start of treatment was defined as day 0. Spheroids were treated with APA-mTS, ETP-mTS, and APA/ETP-mTS micelles every two days, and live-cell-associated fluorescence signals were monitored using IVIS imaging on treatment days 0, 2, 4, 7, and 10. Spheroid growth inhibition was quantitatively assessed using total flux values obtained from the IVIS images (Figure 7).

The inhibitory effect increased over time, reaching reductions of 43.7 ± 5.03%, 64.3 ± 3.21%, and 79.7 ± 17.9%, respectively, by treatment day 10. In contrast, the untreated control group showed an approximately 150% increase in total flux over the same period, indicating continued spheroid growth.

At the final evaluation point, APA/ETP-mTS showed the greatest spheroid growth inhibition among the tested micellar formulations, with total flux approximately 7.39-fold lower than that of the untreated control group (*** *p* < 0.001). These results suggest that the dual-drug micellar formulation effectively suppressed the growth of HeyA8-MDR tumor spheroids under the tested treatment schedule.

### 3.9. In Vivo Toxicity Test

**Figure 8:**
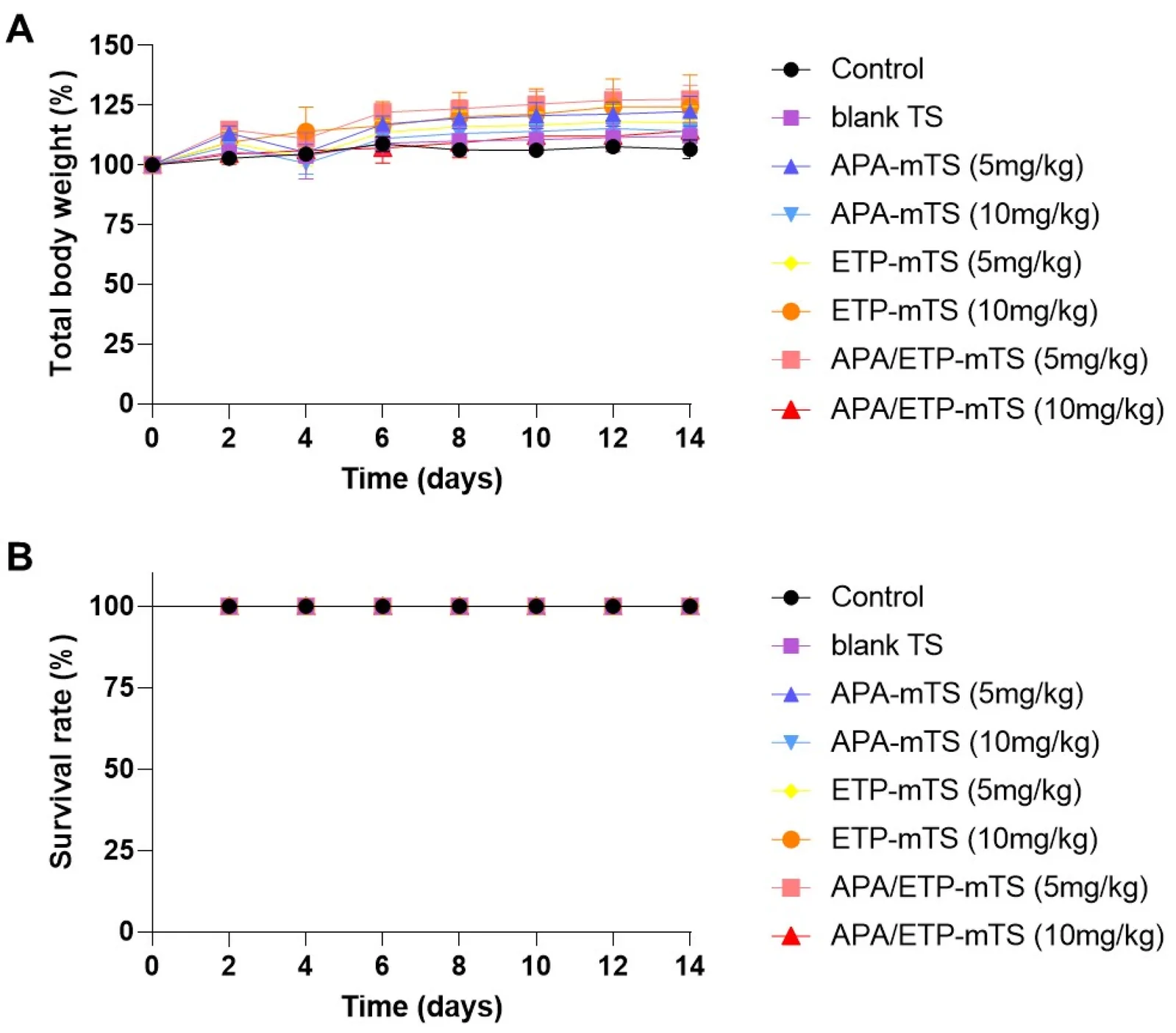
In vivo toxicity evaluation of blank and drug-loaded mTS in ICR mice. (**A**) Relative body weight changes and (**B**) survival rates following repeated intravenous administration of each formulation (n = 6; Mean ± SD).

In vivo toxicity was evaluated in ICR mice by monitoring body weight changes and survival rates after repeated intravenous administration of blank and drug-loaded mTS (Figure 8). No treatment group exhibited body weight loss exceeding 20% of the initial body weight. In addition, no mortality was observed, and survival remained 100% in all groups throughout the observation period. These results suggest that blank mTS and drug-loaded mTS did not induce apparent overt toxicity under the present dosing conditions. The inclusion of the blank mTS group provided preliminary in vivo toxicity information for the TPGS/SOL micellar carrier. Therefore, 10 mg/kg was selected as the final dose for the subsequent in vivo antitumor efficacy study.

### 3.10. In Vivo Antitumor Efficacy

**Figure 9:**
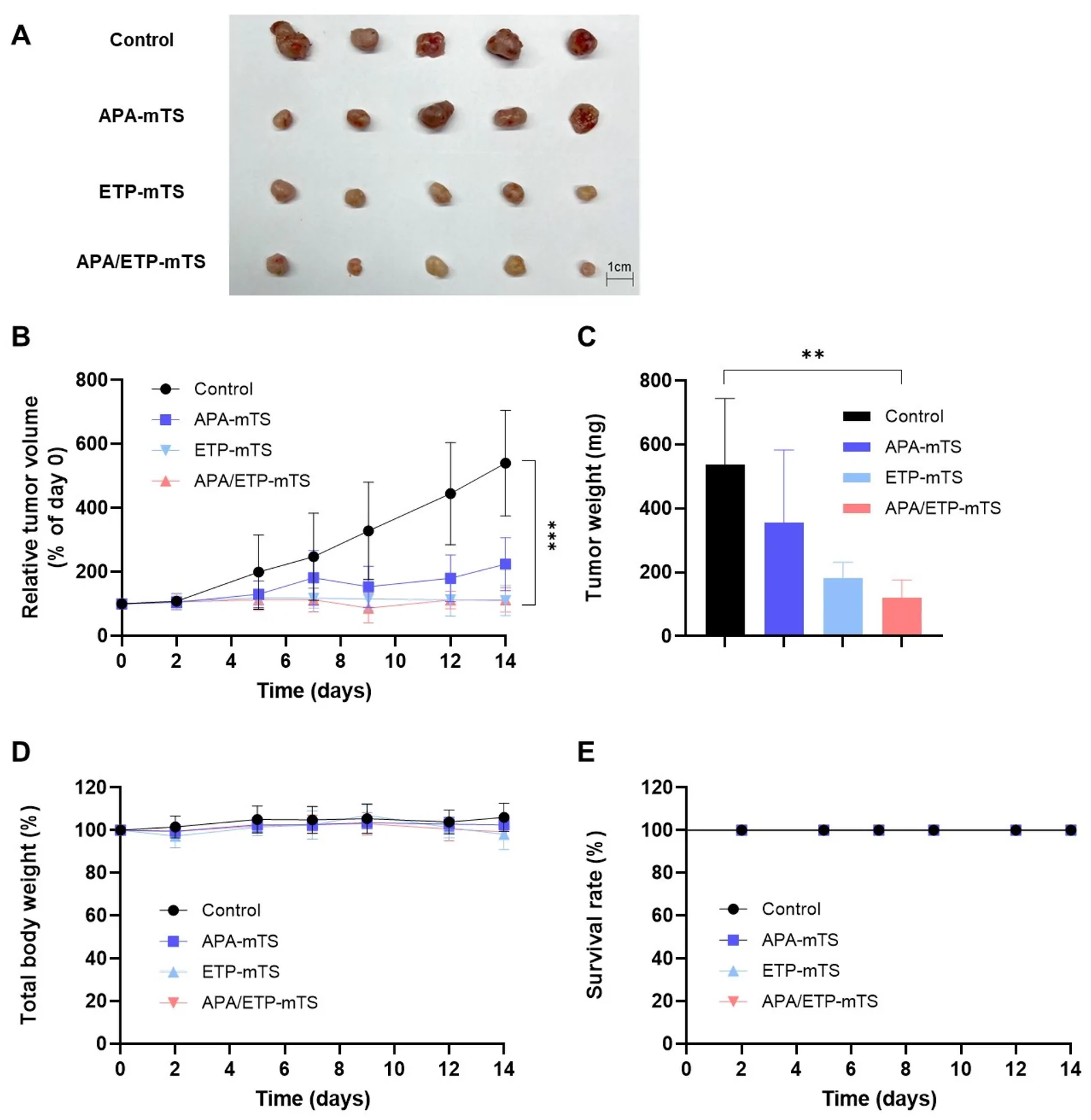
In vivo antitumor efficacy of TS micellar formulations in the HeyA8-MDR subcutaneous xenograft model. (**A**) Images of excised tumor morphology, (**B**) tumor volume changes, (**C**) excised tumor weight, (**D**) body weight changes, and (**E**) survival rates. (** *p* < 0.01, *** *p* < 0.001), (n = 5; Mean ± SD).

The antitumor activity of APA-mTS, ETP-mTS, and APA/ETP-mTS was evaluated in a subcutaneous HeyA8-MDR xenograft mouse model (Figure 9). All drug-loaded micellar formulations inhibited tumor growth compared with the untreated control group. On treatment day 14, tumor volumes in the APA-mTS, ETP-mTS, and APA/ETP-mTS groups were approximately 2.41-, 4.89-, and 4.97-fold lower than those in the control group, respectively.

Consistent with the tumor volume results, excised tumor weights were reduced following treatment with drug-loaded micelles. The tumor weights were 538.02 ± 173.70 mg in the control group, 356 ± 159 mg in the APA-mTS group, 181 ± 40.3 mg in the ETP-mTS group, and 121 ± 45 mg in the APA/ETP-mTS group. The lowest tumor weight was observed in the APA/ETP-mTS treated group. No significant body weight loss or reduction in survival was observed in any treatment group during the efficacy study.

### 3.11. Tumor H&E Staining

**Figure 10:**
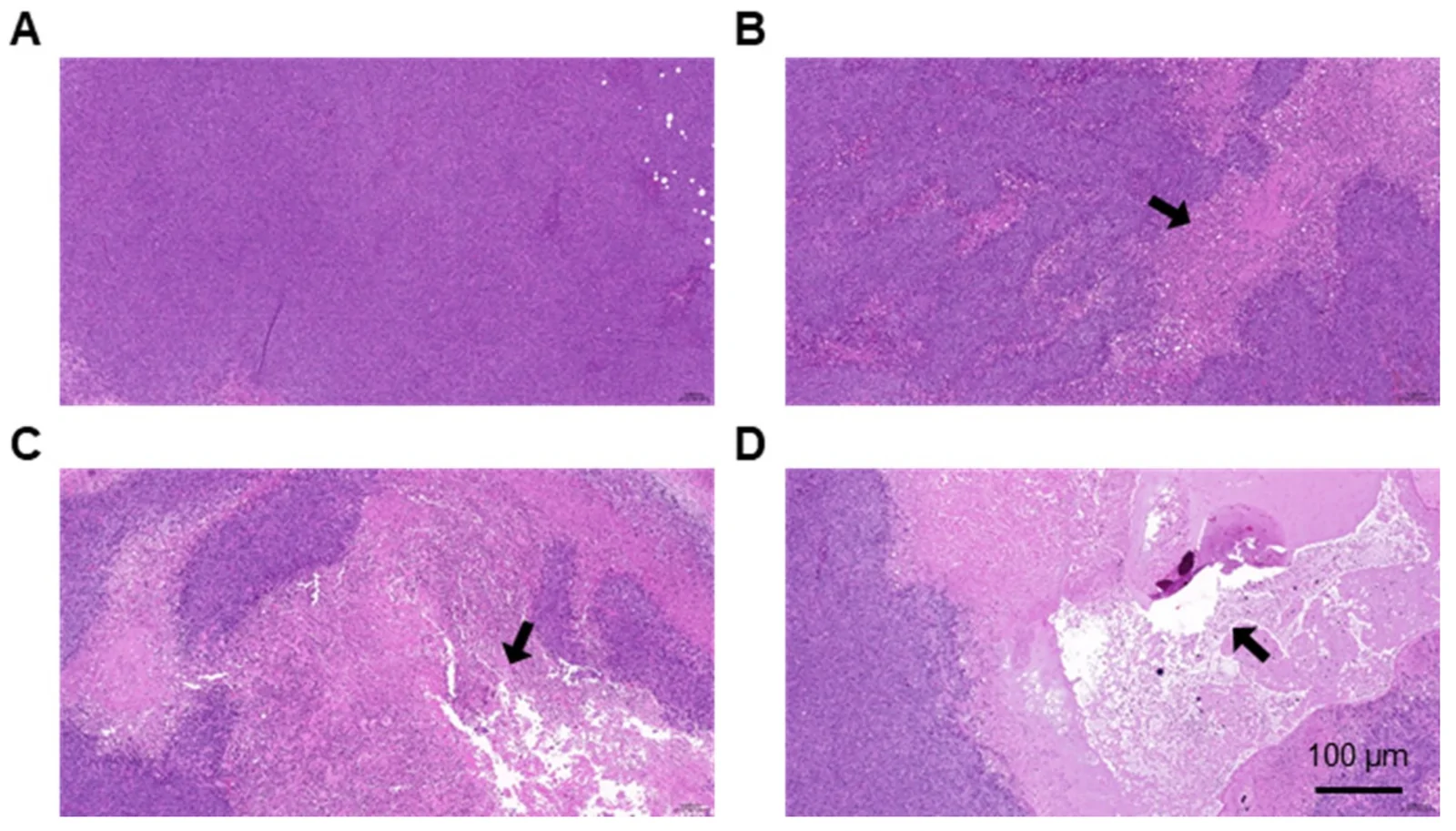
Representative H&E-stained tumor tissue sections from the HeyA8-MDR xenograft model. (**A**) Untreated control, (**B**) APA-mTS, (**C**) ETP-mTS, and (**D**) APA/ETP-mTS groups. Black arrows indicate representative necrotic or disrupted tumor regions.

H&E staining was performed to examine histological changes in tumor tissues after treatment (Figure 10). The control group showed dense tumor cell populations with relatively intact tumor architecture. In contrast, APA-mTS treated tumors showed partial histological disruption, while ETP-mTS treated tumors exhibited more pronounced necrotic regions. The APA/ETP-mTS treated group showed extensive necrotic areas and marked disruption of tumor tissue architecture. These histological observations were consistent with the tumor growth inhibition and reduced tumor weight observed in the in vivo antitumor efficacy study.

## 4. Discussion

In this study, APA/ETP-mTS were developed as a micellar co-delivery system to improve drug delivery and antitumor activity in MDR ovarian cancer models. The formulation was designed to co-encapsulate APA, a VEGFR-2 inhibitor, and ETP, a topoisomerase II inhibitor, within TPGS/SOL mixed micelles. The combined use of TPGS and SOL was intended to improve aqueous dispersion, micellar stability, and cellular accumulation of hydrophobic anticancer drugs. Overall, the results suggest that APA/ETP-mTS enhanced drug co-delivery and tumor growth inhibition in HeyA8-MDR models under the tested conditions [59].

First, HeyA8-MDR cells showed reduced sensitivity to APA and ETP compared with parental HeyA8 cells. The IC_50_ values of APA and ETP were 4.93-fold and 6.40-fold higher, respectively, in HeyA8-MDR cells than in parental HeyA8 cells, indicating that the HeyA8-MDR cells retained a resistant phenotype [60]. Synergy analysis further showed that APA and ETP exhibited synergistic interactions across all tested ratios, supporting their suitability as a combination pair [40]. Among the tested ratios, the 1:1 APA:ETP weight ratio, corresponding to an APA:ETP molar ratio of 1.48:1, was selected as the most suitable ratio for APA/ETP-mTS preparation based on an overall consideration of synergistic interaction, practical dose design, balanced dual-drug loading, and the physicochemical properties of the resulting micellar formulation. In particular, this ratio was compatible with equivalent APA and ETP loading during micelle preparation and with the subsequent in vivo dosing regimen. Therefore, this ratio was used for the preparation of APA/ETP-mTS in the following experiments.

Physicochemical characterization showed that APA/ETP-mTS had nanoscale particle size, low PDI, near-neutral zeta potential, and acceptable encapsulation efficiencies for both drugs. These results indicate that the TPGS/SOL mixed micelle system enabled the successful co-encapsulation of APA and ETP while maintaining a uniform nanosized formulation. During storage at 4 °C, the micelles maintained their particle size, PDI, and zeta potential, although partial decreases in APA and ETP encapsulation efficiencies were observed [56]. Additional stability assessment at 37 °C showed particle size enlargement, increased PDI, and changes in EE values, indicating reduced stability under elevated-temperature conditions. These findings further support refrigerated storage as the preferred condition for APA/ETP-mTS [61].

Although solid-state analyses such as DSC, PXRD, FTIR, or Raman spectroscopy would provide additional information on the physical state and molecular interactions of APA and ETP within the micelles, the present study primarily focused on formulation-performance parameters that are directly related to drug delivery behavior, including EE, DL, storage stability, and in vitro release kinetics. In particular, the sustained-release profiles and release-kinetic fitting provided functional evidence of drug retention and release behavior within the mixed micellar system. These data, together with the physicochemical characterization results, support the successful co-encapsulation and delivery performance of APA/ETP-mTS.

The in vitro drug release study demonstrated formulation-dependent release behavior. Compared with the APA/ETP solution, APA/ETP-mTS delayed the release of both drugs, particularly during the early and intermediate phases. In the dual-drug APA/ETP-mTS micelles, APA showed more sustained release than ETP, suggesting prolonged retention of APA within the mixed micellar system [62,63]. The release profile differed between solution and dual-drug micelles, which may be related to altered drug–polymer interactions or changes in micellar core packing after dual-drug loading [64,65]. The release experiment was performed in PBS at pH 7.4 to evaluate the baseline sustained-release and drug-retention behavior of APA/ETP-mTS under the selected physiological buffer condition. Similar pH 7.4 release conditions have been used in previous studies of TPGS/Soluplus^®^ mixed micelles and APA- or ETP-loaded nanocarriers to assess formulation-dependent release behavior [28,66,67,68,69].

C6-based fluorescence analysis showed that C6-mTS micelles produced higher intracellular fluorescence intensity than C6-mSOL micelles in HeyA8-MDR cells, suggesting that TPGS incorporation was associated with enhanced cellular accumulation. This observation is consistent with previous reports describing P-gp-modulatory activity of TPGS; however, because direct P-gp expression or transport activity was not measured, P-gp modulation should be interpreted as a possible contributing mechanism rather than a confirmed mechanism [70,71,72].

Cytotoxicity and 3D spheroid studies further validated the enhanced therapeutic efficacy of the APA/ETP-loaded micelles. The combination micelle formulation showed lower IC_50_ values compared with free drugs and suppressed spheroid growth more efficiently [71]. This finding supports the contribution of TPGS incorporation to enhanced cytotoxicity [73]. In monolayer HeyA8-MDR cells, ETP-mTS showed the lowest IC_50_ among the tested formulations, whereas APA/ETP-mTS did not show superior IC_50_ compared with ETP-mTS. This result suggests that the benefit of APA/ETP-mTS should not be interpreted solely based on 2D monolayer IC_50_ values. Rather, APA/ETP-mTS showed biological activity across multiple models, including marked spheroid growth inhibition and reduced tumor weight in the xenograft model, suggesting that co-delivery may provide advantages under more complex tumor conditions [74]. These findings suggest that APA/ETP-mTS retained cytotoxic activity and showed enhanced performance in the tumor spheroid model, highlighting the inhibitory effect on MDR tumor growth [75].

The spheroid and xenograft studies used the same repeated-treatment interval of every two days, although IVIS imaging and tumor-volume measurements were performed at model-appropriate observation points. Spheroids were monitored by IVIS until treatment day 10, whereas xenograft tumors were followed until treatment day 14, reflecting model-specific experimental feasibility.

In the HeyA8-MDR subcutaneous xenograft model, APA/ETP-mTS showed antitumor activity among the tested micellar formulations [58,76]. Tumor volume in the APA/ETP-mTS group was approximately 4.97-fold lower than that in the untreated control group on treatment day 14, and the lowest excised tumor weight was observed in this group. Although ETP-mTS and APA/ETP-mTS showed comparable tumor volume suppression, APA/ETP-mTS resulted in the lowest final tumor weight. However, because free APA/ETP combination and TPGS-free APA/ETP-mSOL controls were not included in the in vivo efficacy study, these results should be interpreted as comparative efficacy among the tested micellar formulations rather than as definitive evidence of superiority over all relevant control formulations. Repeated administration did not cause body weight loss exceeding 20% or mortality, suggesting no apparent overt toxicity under the tested dosing conditions. H&E staining further showed extensive necrotic areas and disruption of tumor tissue architecture in APA/ETP-mTS treated tumors, consistent with the in vivo tumor inhibition results. These antitumor effects are consistent with the established pharmacological activity of APA as a VEGFR-2 tyrosine kinase inhibitor, which has been reported to suppress VEGF/VEGFR-2-related angiogenic signaling and downstream survival pathways [10,11,12]. Therefore, the tumor growth inhibition observed after APA/ETP-mTS treatment may be partly associated with the VEGFR-2-targeting activity of APA, together with improved micellar co-delivery of APA and ETP. Because the APA/ETP-mTS-treated tumors were markedly reduced in size and contained extensive necrotic regions, the amount of residual viable tumor tissue suitable for matched immunohistochemical analyses across all groups was limited. Therefore, H&E staining was prioritized to evaluate overall tumor morphology and tissue-level treatment response. Additional VEGFR-2 pathway analysis and apoptosis- or proliferation-related staining, such as TUNEL and Ki67, would further refine the mechanistic interpretation of these antitumor effects.

The blank mTS group included in the in vivo toxicity study provided preliminary evidence for the absence of marked carrier-associated toxicity under the tested dosing conditions, as no marked body weight loss or mortality was observed. In addition, TPGS and Soluplus^®^ are widely used pharmaceutical excipients for micellar drug delivery, and previous mTS studies have reported the use of blank mTS in HeyA8 ovarian cancer cells and the absence of marked toxicity in vivo [28,32,63,70]. The absence of marked toxicity of blank TPGS/Soluplus^®^-based formulations in non-cancer cells has also been reported [77]. Collectively, these findings support the absence of marked toxicity associated with the TPGS/Soluplus^®^ carrier under the tested in vivo conditions and provide supporting background for the safety of this carrier platform.

Overall, APA/ETP-mTS improved drug co-delivery and enhanced tumor growth inhibition in MDR ovarian cancer models under the tested conditions. These findings support the potential of TPGS/SOL mixed micelles as a promising co-delivery platform for hydrophobic anticancer agents in resistant ovarian cancer. Given the broad utility of micellar systems for improving the delivery of poorly water-soluble drugs, this platform may also have potential applicability to other ovarian cancer cell types and, more broadly, to other cancer models requiring improved intracellular delivery of hydrophobic anticancer agents.

## 5. Conclusions

In this study, APA and ETP were successfully co-encapsulated into TPGS/SOL mixed micelles to improve aqueous dispersion, cellular accumulation, and antitumor activity in MDR ovarian cancer models. The resulting APA/ETP-mTS formulation showed nanoscale particle size, low PDI, acceptable encapsulation efficiencies, sustained drug release, and improved cellular accumulation compared with TPGS-free micelles. APA/ETP-mTS also showed growth-inhibitory activity in HeyA8-MDR tumor spheroids and suppressed tumor growth in the HeyA8-MDR xenograft model under the tested treatment conditions. Repeated administration did not induce apparent overt toxicity based on body weight and survival observations. Overall, these findings suggest that APA/ETP-mTS is a promising micellar co-delivery platform for hydrophobic anticancer drugs in MDR ovarian cancer models, with potential applicability to other ovarian cancer cell types and broader cancer models requiring improved delivery of poorly water-soluble anticancer agents. Future mechanistic, pharmacokinetic, biodistribution, and safety studies will further support its translational development.

## 6. Patents

A Korean patent application related to this work has been filed (Application No. 10-2026-0011893).

## Figures and Tables

**Table 1:** Physicochemical properties of APA/ETP-mTS prepared by thin-film hydration using different TPGS:SOL polymer compositions.

TPGS:SOL Weight Ratio	Amount of Polymer Used (mg)	Amount of Drug Used (mg)		Encapsulation Efficiency (EE, %)		Drug Loading Capacity (DL, %)		Particle Size (nm)	Poly-Dispersity Index (PDI)	Zeta Potential (mV)
		APA	ETP	APA	ETP	APA	ETP			
1:1	100	5	5	47.7 ± 1.56	47.6 ± 6.57	2.28 ± 0.07	2.27 ± 0.31	15.8 ± 2.55	0.13 ± 0.05	4.35 ± 1.35
2:1	100	5	5	57.7 ± 3.37	54.7 ± 3.13	2.73 ± 0.16	2.59 ± 0.15	18.0 ± 6.26	0.15 ± 0.04	−0.67 ± 0.41
3:1	100	5	5	63.5 ± 0.55	65.2 ± 1.18	2.98 ± 0.03	3.06 ± 0.06	20.0 ± 5.05	0.16 ± 0.05	−0.93 ± 0.97

**Table 2:** Korsmeyer–Peppas release kinetic parameters of APA and ETP.

Drug	Formulation	K (%·h^−n^)	n	R^2^
APA	APA/ETP solution	32.6	0.169	0.985
	APA/ETP-mTS	8.61	0.325	0.919
ETP	APA/ETP solution	41.6	0.152	0.996
	APA/ETP-mTS	11.5	0.363	0.993

## Data Availability

The data presented in this study are available within the article.

## Supplementary Materials

The following supporting information can be downloaded at: https://www.mdpi.com/article/10.3390/pharmaceutics18091143/s1, Figure S1. In vitro cytotoxicity of free APA and ETP in parental HeyA8 and HeyA8-MDR cells. Dose-response curves of (**A**) APA in HeyA8 cells, (**B**) APA in HeyA8-MDR cells, (**C**) ETP in HeyA8 cells, and (**D**) ETP in HeyA8-MDR cells. The HeyA8-MDR free-drug datasets in panels B and D are also presented in Figure 5A,B as free-drug controls for comparison with micellar formulations. Data are presented as the mean ± SD (n = 6); Figure S2. Representative TEM image of APA/ETP-mTS. The image show nanosized micellar structures and provide qualitative morphological support for the DLS-based particle size analysis; Figure S3. Stability evaluation of APA/ETP-mTS at 37 °C. Changes in (**A**) APA EE, (**B**) ETP EE, (**C**) particle size, (**D**) PDI, and (**E**) zeta potential were monitored during storage (n = 3; Mean ± SD).

## Abbreviations

The following abbreviations are used in this manuscript:

APA	Apatinib
ETP	Etoposide
TPGS	D-α-tocopheryl polyethylene glycol succinate
SOL	Soluplus^®^
MDR	Multidrug resistance
P-gp	P-glycoprotein
TS	TPGS/SOL mixed micelles
TKI	Tyrosine kinase inhibitor
CMC	Critical micelle concentration
C6	Coumarin-6
MTT	Thiazolyl blue tetrazolium bromide
MeOH	Methanol
DW	Distilled water
NaOH	Sodium hydroxide
ACN	Acetonitrile
TEA	Triethylamine
HPLC	High-performance liquid chromatography
FBS	Fetal bovine serum
RPMI-1640	Roswell Park Memorial Institute 1640 medium
DPBS	Dulbecco’s phosphate-buffered saline
DLS	Dynamic light scattering
PDI	Polydispersity index
CI	Combination index
EE	Encapsulation efficiency
IC_50_	Half-maximal inhibitory concentration
Topo II	Topoisomerase II
VEGFR-2	Vascular endothelial growth factor receptor-2
PBS	Phosphate-buffered saline
DMSO	Dimethyl sulfoxide
PEG	Polyethylene glycol
IACUC	Institutional Animal Care and Use Committee
H&E	Hematoxylin and eosin
IVIS	In vivo imaging system
ULA	Ultra-low attachment
