# Supplementary material for: Antitumor Efficacy of Apatinib and Etoposide-Loaded D-α-Tocopheryl Polyethylene Glycol Succinate Mixed Micelles Against Multidrug-Resistant Ovarian Cancer Cells
Source: Pharmaceutics. 2026 Sep 10;18(9):1143. doi: 10.3390/pharmaceutics18091143 (PMC13610361; doi:10.3390/pharmaceutics18091143)
Supplement: Supplementary file 1 [file pharmaceutics-18-01143-s001.zip › pharmaceutics-4526102-supplementary.pdf]

pharmaceutics

MDPI

Article

Antitumor Efficacy of Apatinib and Etoposide-Loaded D-α-Tocopheryl Polyethylene Glycol Succinate Mixed Micelles Against Multidrug-Resistant Ovarian Cancer Cells

Myeong Kyun Yoo[1†], Su Jeong Kang[1†], Min Jeong Jo[1], Jae Min Lee[1], Moon Sup Yoon[1], Seon Min Park[1], Sinem Yaprak Karavana[2], and Dae Hwan Shin[1,3,4,*]

1 College of Pharmacy, Chungbuk National University, Cheongju 28160, Republic of Korea
2 Department of Pharmaceutical Technology, Faculty of Pharmacy, Ege University, Bornova, Izmir, Turkey
3 Korea Animal Replacement Experiment Co., Ltd. (KARE), Cheongju, 28160, Republic of Korea
4 Chungbuk National University Hospital, Chungbuk National University, Cheongju, 28644, Republic of Korea
* Correspondence: dshin@chungbuk.ac.kr; Tel.: +82-43-261-2820

Supplementary Materials:

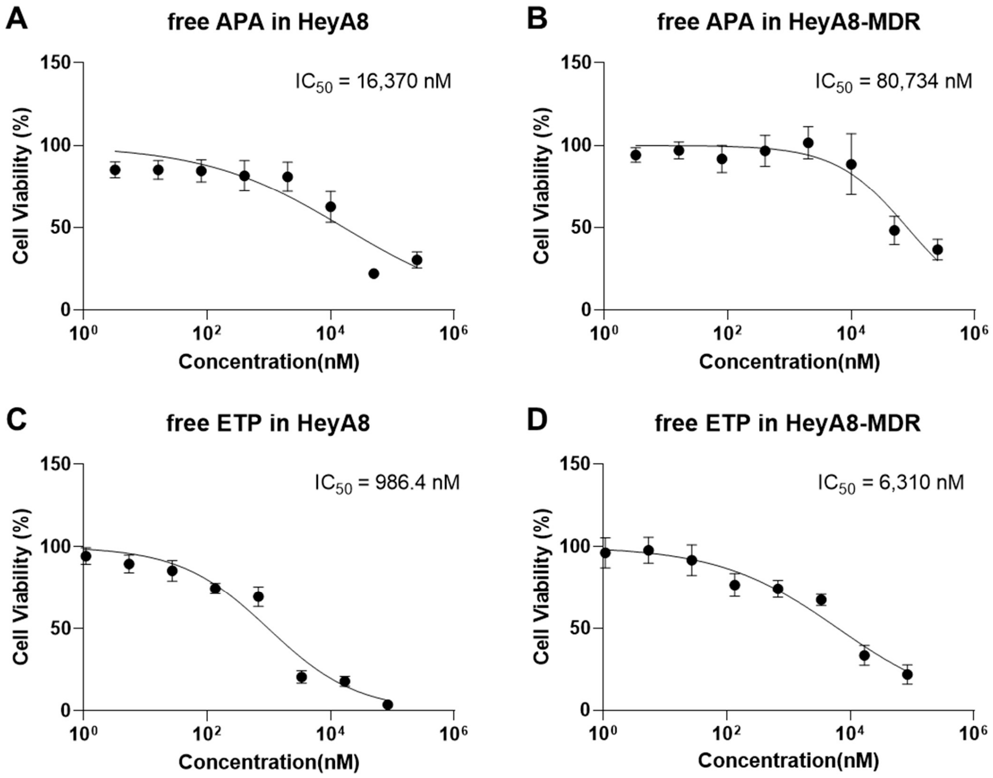

Figure S1. *In vitro* cytotoxicity of free APA and ETP in parental HeyA8 and HeyA8-MDR cells. Dose-response curves of (A) APA in HeyA8 cells, (B) APA in HeyA8-MDR cells, (C) ETP in HeyA8 cells, and (D) ETP in HeyA8-MDR cells. The HeyA8-MDR free-drug datasets in panels B and D are also presented in Figure 5A and 5B as free-drug controls for comparison with micellar formulations. Data are presented as the mean ± SD (n = 6).

 https://doi.org/10.3390/pharmaceutics18091143

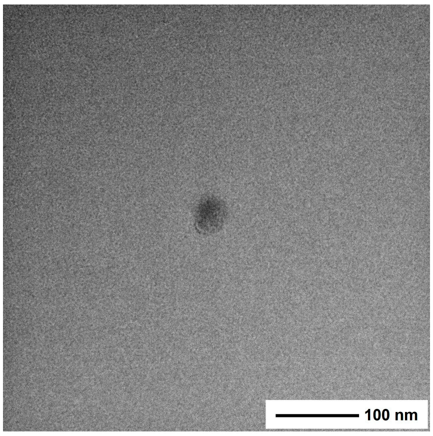

Figure S2. Representative TEM image of APA/ETP-mTS. The image show nanosized micellar structures and provide qualitative morphological support for the DLS-based particle size analysis.

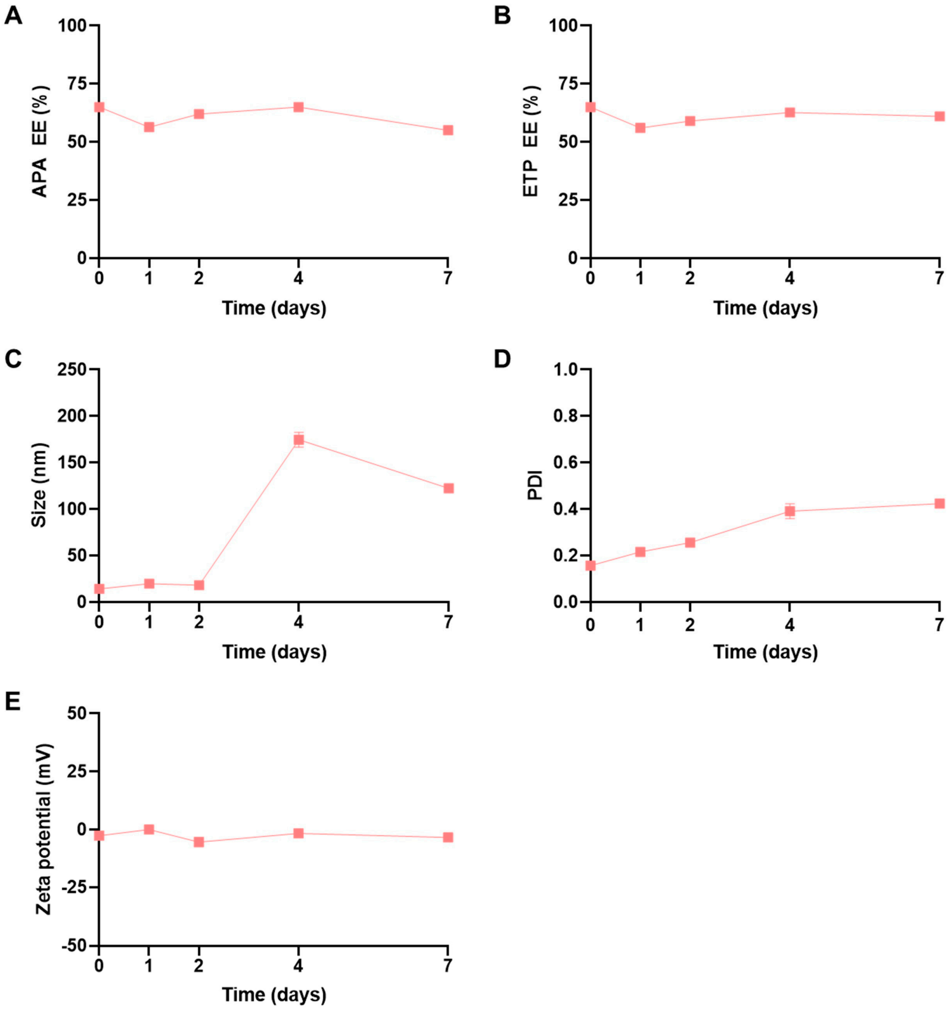

Figure S3. Stability evaluation of APA/ETP-mTS at 37 °C. Changes in (A) APA EE, (B) ETP EE, (C) particle size, (D) PDI, and (E) zeta potential were monitored during storage (n = 3; Mean ± SD).
